# Temporal stability and molecular persistence of the bone marrow plasma cell antibody repertoire

**DOI:** 10.1038/ncomms13838

**Published:** 2016-12-21

**Authors:** Gabriel C. Wu, Nai-Kong V. Cheung, George Georgiou, Edward M. Marcotte, Gregory C. Ippolito

**Affiliations:** 1 Center for Systems and Synthetic Biology, University of Texas at Austin, Austin, Texas 78712, USA; 2 Department of Pediatrics, Memorial Sloan-Kettering Cancer Center, New York, New York 10065, USA; 3 Department of Molecular Biosciences, University of Texas at Austin, Austin, Texas 78712, USA; 4 Department of Biomedical Engineering, University of Texas at Austin, Austin, Texas 78712, USA; 5 Department of Chemical Engineering, University of Texas at Austin, Austin, Texas 78712, USA; 6 Institute for Cell and Molecular Biology, University of Texas at Austin, Austin, Texas 78712, USA

## Abstract

Plasma cells in human bone marrow (BM) are thought to be responsible for sustaining lifelong immunity, but its underlying basis is controversial. Here we use high-throughput sequence analysis of the same individual across 6.5 years to show that the BM plasma cell immunoglobulin heavy chain repertoire is remarkably stable over time. We find a nearly static bias in individual and combinatorial gene usage across time. Analysis of a second donor corroborates these observations. We also report the persistence of numerous BM plasma cell clonotypes (∼2%) identifiable at all points assayed across 6.5 years, supporting a model of serological memory based upon intrinsic longevity of human plasma cells. Donors were adolescents who completely recovered from neuroblastoma prior to the start of this study. Our work will facilitate differentiation between healthy and diseased antibody repertoires, by serving as a point of comparison with future deep-sequencing studies involving immune intervention.

## Abstract (web summary)

Longevity of antibody responses has been attributed to persistence of plasma cells in mice. Here the authors provide human data in support of this model by immunoglobulin sequencing bone marrow sections from two human donors over 6.5 years to show temporal stability of plasma cell clonotypes, but not other B cells.

The human bone marrow (BM) is a specialized immune compartment that is responsible for both the initial generation of newly formed B cells and the maintenance of terminally differentiated, antibody-secreting plasma cells. The BM, and the plasma cells it harbours, is a central site of antibody production and is the major source of all classes and subclasses of human immunoglobulins (Ig) in the serum^1^,^2^. Ig-secreting BM plasma cells are generally believed to be ‘long-lived' and to persist for the lifespan of the organism^3^. Longitudinal serological studies have established that antiviral serum antibodies can be remarkably stable, with half-lives ranging from 50 years (for example, varicella-zoster virus) to 200 years (for example, measles and mumps); however, by contrast, antibody responses to non-replicating antigens (for example, tetanus and diphtheria bacterial toxins) rapidly decay with much shorter half-lives of only 10–20 years^4^. These differences not only suggest that antigen-specific mechanisms have a substantial role in the establishment and/or maintenance of serological memory, but raises the question of whether the differential stability of antibody responses might reflect differential intrinsic longevity of plasma cells. This mechanism has been previously proposed in the context of vaccinations and infections^4^,^5^, and is also supported by observations of differential stability of autoantibody titers when using B-cell depleting therapies to treat autoimmune diseases^6^,^7^.

The basis of lifelong serological memory (antibody responses) is controversial^3^,^8^,^9^. A model for intrinsic longevity in plasma cell survival (and hence longevity in serum antibody maintenance) has been posited for the laboratory mouse^10^,^11^, but data for human plasma cells have not been generated. On the basis of mouse models, human BM plasma cells are assumed to be similarly long-lived and the major source of serum antibodies; however, the contribution of antigen-specific BM plasma cells in humans has only recently been shown experimentally^5^,^12^. Despite these notable advances, the availability of corresponding molecular data (namely, sequence data of BM plasma cell Ig transcripts) and of information regarding plasma cell dynamics *in vivo* is scarce. Persistent antigens as well as the memory B-cell compartment are implicated in alternative models of lifelong serological memory, implying continual clonal replacement of antigen-specific plasma cells, in contrast to intrinsic plasma cell longevity^13^,^14^,^15^.

Three studies have generated BM plasma cell data using next-generation sequencing techniques, but did not examine the temporal changes that occur in the antibody repertoire over time^5^,^16^,^17^. Here, building upon our prior experiences with the comprehensive analysis of human cellular and serological antibody repertoires^18^,^19^,^20^,^21^,^22^, we present the first longitudinal study of serially acquired human BM plasma cells assayed by next-generation deep sequencing. To directly measure the temporal dynamics of BM plasma cells—and to indirectly gain insight into long-lived serological memory—we sequence recombined VHDJH regions (cDNA), which encode the variable domain (protein) of antibody IGH heavy chains. Most of the VHDJH genetic diversity is in the CDR-H3 hypervariable interval (encoded by a D element, random non-templated nucleotides, and small portions of the VH and JH elements). CDR-H3 is a primary determinant of antibody specificity^23^,^24^ and has long been considered a unique ‘fingerprint' which aids identification of a progenitor B cell and its clonal progeny (B-cell clonotype)^25^. We sequence BM plasma cells from the same individual at seven time points over a total of 6.5 years and from a second individual with two time points over 2.3 years. The temporal resolution and duration of sampling provides a method to interrogate the *in vivo* temporal dynamics of BM plasma cells in a previously uncharacterized way. We provide detailed temporal information on the individual genes (IGH V, D and J), gene combinations (V-D, V-J, D-J, V-D-J) and temporally persistent CDR-H3 clonotypes. The second individual provides support that our observations are not unique. Moreover, persisting CDR-H3 clonotypes are class-switched and somatically mutated (in the IGHV gene segment) implying derivation from activated B-cell progenitors that must have been selected by antigen. Crucially, persisting CDR-H3 clonotypes reside exclusively in the plasma cell compartment, but are absent among comparable memory B cells (mBCs) (also a class-switched and somatically mutated B-cell compartment) isolated from the same BM biopsy. Overall, our results underscore the temporal stability of the IGH V region repertoire according to multiple metrics (temporally stable IGH molecular phenotypes), and provide unequivocal sequence-based evidence for the persistence of plasma cell clonotypes spanning 6.5 years.

## Results

### Next-generation sequencing of serial BM biopsies

**Figure 1:**
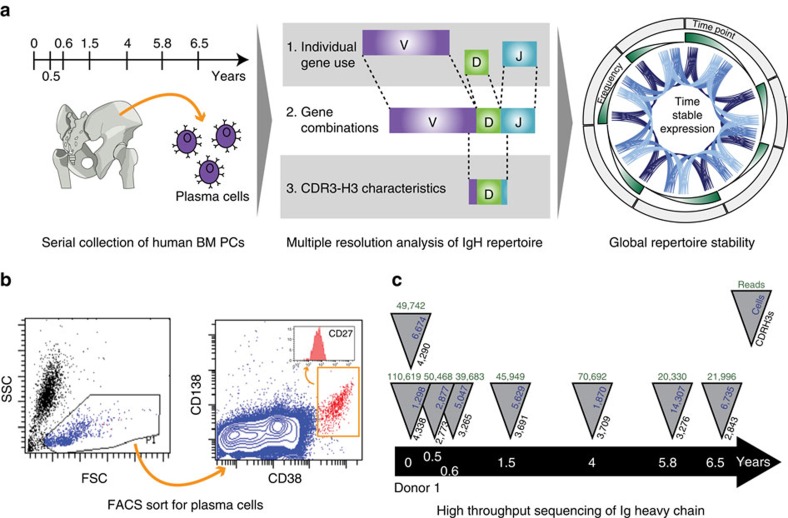
Overview of bone marrow plasma cell sampling and NGS. (**a**) Overview of antibody repertoire characterization method. Serial sampling of human bone marrow (BM) plasma cells over 6.5 years (left). Analysis of individual genes, gene combinations and CDR-H3s (centre) show temporally stable expression of persistent entities (right). (**b**) Representative fluorescence-activated cell sorting (FACS) gates of BM plasma cells (CD138+, CD38++) isolated from bone marrow mononuclear cells (BMMCs). (**c**) Sample collection timeline and summary of cell counts, quality-filtered sequencing reads and unique CDR-H3s for Donor 1.

To investigate the temporal dynamics of the IGH antibody gene repertoire of BM plasma cells, we sampled, sorted and performed high-throughput sequencing (Fig. 1a). Serial BM biopsies were obtained from two adolescents (Supplementary Table 1) as part of routine evaluations for non-immuno-haematological disease. BM plasma cells were isolated using fluorescence-activated cell sorting (FACS) for CD38++ CD138+ cells within the mononuclear light-scatter gate (Fig. 1b). Additionally, the cells were uniformly positive for the tumour-necrosis factor-receptor superfamily member CD27 (Fig. 1b, inset). Importantly, we avoided gating of the pan-B-cell marker CD19 since previous characterizations of human BM plasma cells show heterogeneous expression of CD19 (refs 26, 27). Therefore, our method captured all recently described BM plasma cell subpopulations^5^,^12^ with an overall CD19^+/−^ CD27^+^ CD38^++^ CD138^+^ phenotype. Subsequently, transcripts were amplified from BM plasma cells expressing IgM, IgG and IgA using polymerase chain reaction with reverse transcription (RT-PCR) followed by high-throughput sequencing.

In total, 503,415 total sequencing reads were generated from 51,200 BM plasma cells (see Methods and Supplementary Table 1). These data span seven time points across 6.5 years for Donor 1 (Fig. 1c) and two time points across 2.3 years for Donor 2. A biological replicate (that is, a second frozen ampule derived from the same BM aspiration) was also collected from each donor and analysed. Replicate sampling from the same donor and time point allowed us to confidently discern the active spectrum of heavy chain genes comprising a donor's antibody repertoire.

### Individual gene frequencies are highly stable

**Figure 2:**
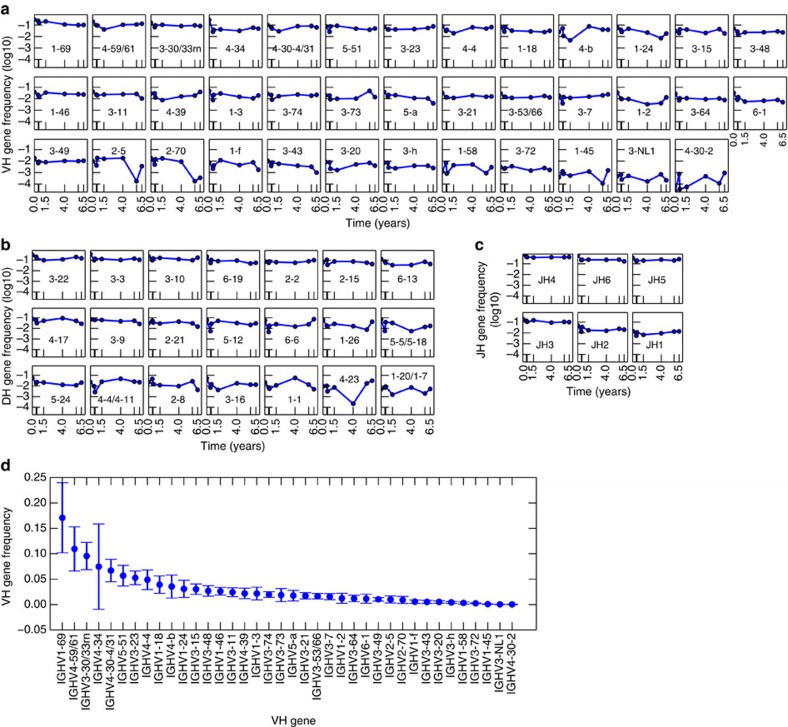
IGH gene segment frequencies among BM plasma cells are temporally stable. For Donor 1: (**a**–**c**) IGHV (**a**), IGHD (**b**) and IGHJ (**c**) gene usage frequency over time. Plots are sorted by decreasing mean frequency. Only gene identifications that appear in all time points are shown. (**d**) Mean frequency of IGHV gene use. Error bars are s.d.

For Donor 1, we identified 38 IGHV genes, 21 IGHD genes and 6 IGHJ genes (4,788 combinations). We assessed the frequency of each IGH V, D and J gene across time (Fig. 2) and found stability of individual gene usage. The most frequently used genes (for example, IGHV4-34) show consistently high expression while less frequently used genes (for example, IGHV3-72) show consistently low expression. This observation was quantified using the Mann-Kendall Test, which evaluates trends in time series data. We find that 89% of IGHV genes, 95% of IGHD genes and 100% IGHJ genes show no statistically significant trends (Mann-Kendall test, *P*>0.05), indicating that the IGHV (Fig. 2a), IGHD (Fig. 2b) and IGHJ (Fig. 2c) genes are time stable.

Next, we analysed population behaviour of gene usage. Averaging across all time points, we observe a highly skewed distribution of individual gene frequencies, consistent with previous single time point observations. Only 6 IGHV genes (16%) account for greater than 50% of total IGHV gene usage by frequency (Fig. 2d). IGHD2-2, IGHD3-3 and IGHD3-22 (ref. 28), previously shown to have biased usage, together account for 33% of total IGHD usage (Fig. 2b). In addition, known biases in IGHJ usage^29^ are recapitulated. IGHJ4, IGHJ6 and IGHJ5 account for 86% of total IGHJ usage. Furthermore, IGH V, D and J gene usage are not significantly different from a log-normal distribution (Anderson-Darling, H=0, *P*>0.05).

### Gene combinations frequencies are stable over time

**Figure 3:**
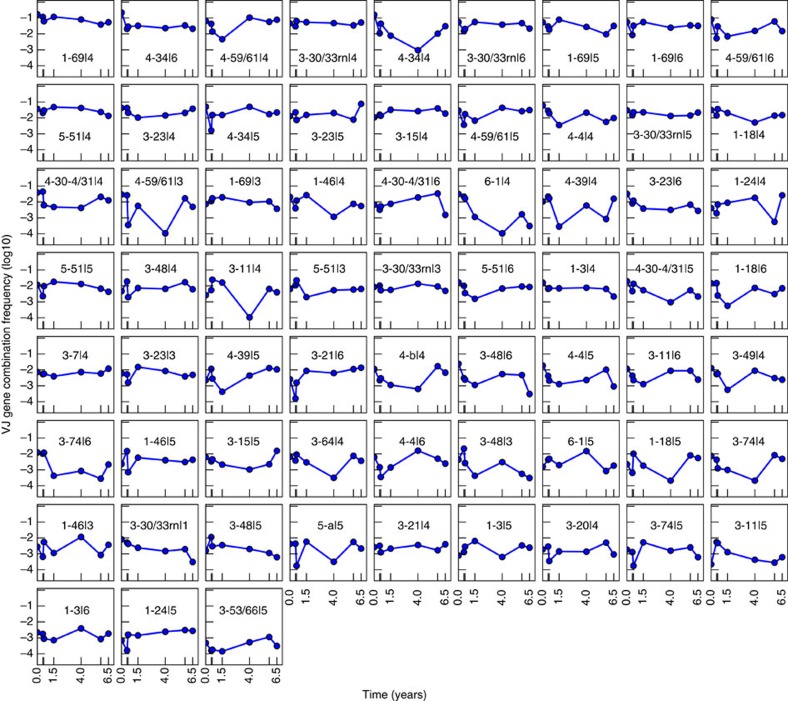
Frequencies of gene combinations among BM plasma cells are temporally stable. IGH V-J usage frequencies for Donor 1 are shown. Plots are sorted by decreasing mean frequency. Only gene identifications that appear in all time points are shown. See Supplementary Fig. 1a–c for usage frequencies of IGH V-D, D-J and V-D-J.

Given the temporal stability of individual genes, we hypothesized that differential intrinsic longevity might be found in gene combinations. Surprisingly, our analysis indicates that gene combinations, like their individual component genes, are time stable as well. We find that 92% V-J (Fig. 3), 97% V-D (Supplementary Fig. 1), 95% D-J (Supplementary Fig. 2) and 97% V-D-J (Supplementary Fig. 3) do not show significant trends (Mann-Kendall, H=0, *P*>0.05).

**Figure 4:**
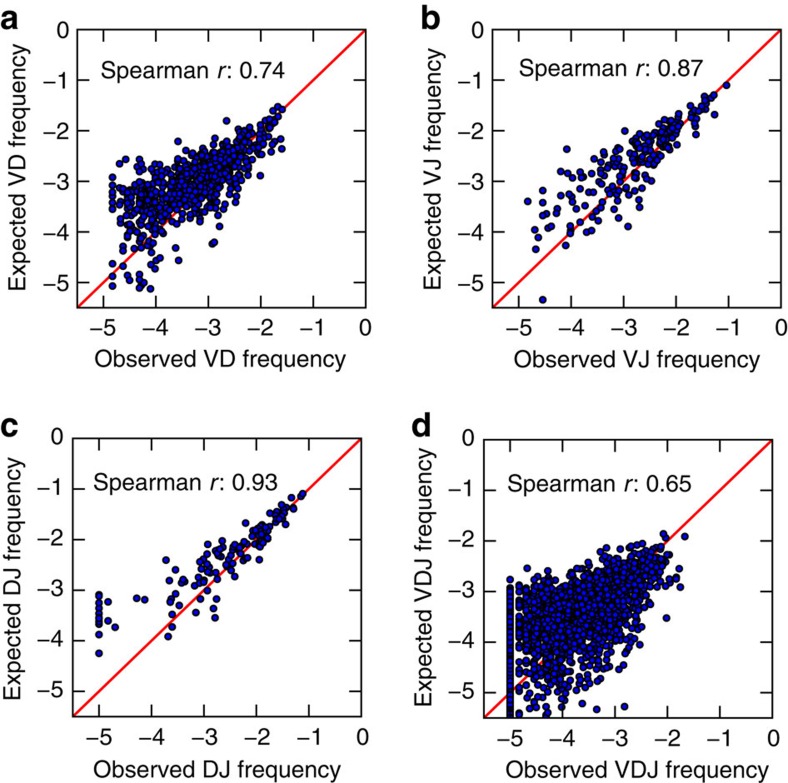
Gene combinations among BM plasma cells do not preferentially associate. Gene combinations are randomly assorted in Donor 1. (**a**–**d**) Spearman's rank correlation of expected versus observed IGH V-D (**a**), V-J (**b**), D-J (**c**) and V-D-J (**d**) gene combination frequencies. Expected (by random association) frequencies are calculated as products of the frequencies of the individual component genes. Diagonal lines in red indicate no difference between the expected and observed frequencies.

To better understand the nature of gene combinations, we analysed preferential gene pairing biases by comparing the expected versus observed frequency of pairwise gene combinations. The observed frequency of each gene combination is correlated to its expected frequency (Spearman *r*): V-D (0.74), V-J (0.87), D-J (0.93) and V-D-J (0.65) (Fig. 4a–d). This high level of correlation and lack of significant outliers suggests minimal gene pairing linkage and that gene pairing is a random process.

### Persistent CDR-H3 clonotypes are unique to BM plasma cells

**Figure 5:**
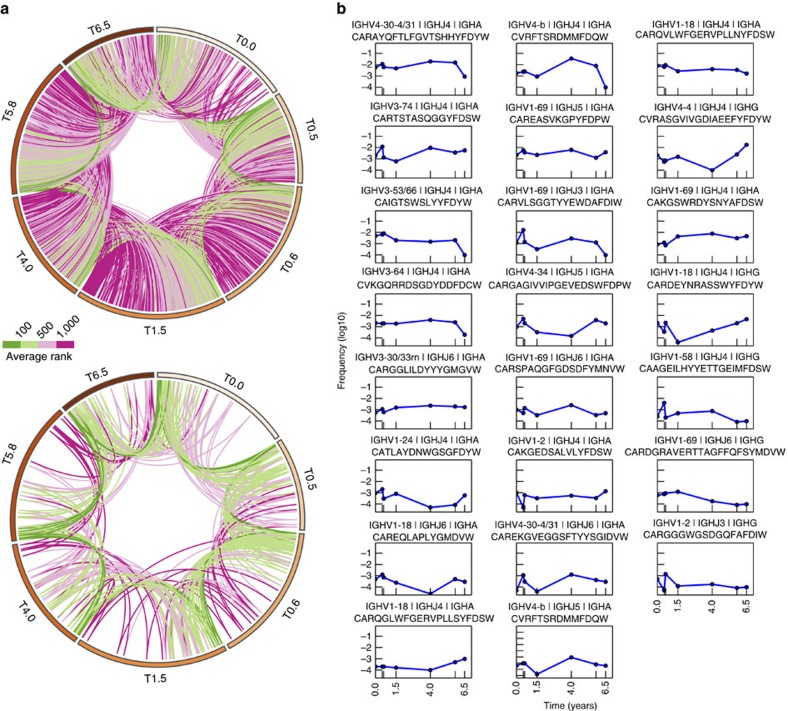
Frequencies of persistent antibody clonotypes among BM plasma cells are temporally stable. (**a**) Circos plot of shared CDR-H3 antibody clonotypes between adjacent time points across 6.5 years for Donor 1 (top). Circos plot of the persistent clonotypes across all time points (bottom). Each band in the outermost perimeter represents the clonotypes found in a given time point, sorted by decreasing frequency. The inner curved lines indicate the same clonotype shared by two time points. Green indicates high frequency; purple, low frequency; with lighter colours indicating intermediate frequency. (**b**) Gene usage frequency over time of the 23 persistent clonotypes (see Methods) found in all time points. Plots are sorted by decreasing mean frequency. Gene names (for IGHV and IGHJ), representative amino acid sequences and isotype are above each plot. (The 165 persistent CDR-H3 antibody clonotypes for Donor 2 are shown in Supplementary Fig. 12.)

To understand how each of these individual genes and gene combinations together might indicate the existence of long lived plasma cells, we analysed the behaviour of the CDR-H3, the highest resolution possible for a single identifier of an antibody producing cell. To eliminate errors and ambiguities, we clustered CDR-H3s into clonotypes based on previously established criteria (see Methods). On average, 16% of clonotypes are shared between adjacent time points (Fig. 5a, top). Comparison of the BM plasma cell compartment with mBCs co-isolated from the same biopsy specimens provided a baseline to gauge stability across the larger framework of the B-cell compartment. In mBCs, gene stability was statistically similar to the plasma cell compartment (Supplementary Fig. 11). However, no persistent CDR-H3 clonotypes were found among 58,953 mBCs isolated by flow cytometry from the same biopsies across four years in this same donor.

Interestingly, among BM plasma cells, 23 clonotypes persist across all time points spanning 6.5 years (Fig. 5b). We find that 100% of these persistent clonotypes are time stable (Fig. 5a, bottom, Mann-Kendall test, h=0, *P*>0.05) and 78% (18/23) are of the IgA isotype. In addition, characteristics of the complete CDR-H3 population, specifically CDR-H3 lengths (Supplementary Fig. 4) and hydropathy index (Supplementary Fig. 5a), are unchanged over time. The overall total distribution of CDR-H3 lengths are consistent with previously reported single time point values. Also, higher expressing CDR-H3s tend to be neither hydrophobic nor hydrophilic (Supplementary Fig. 5b) and we find no significant trends between hydrophobicity and expression level.

### Second donor corroborates observations from first donor

**Figure 6:**
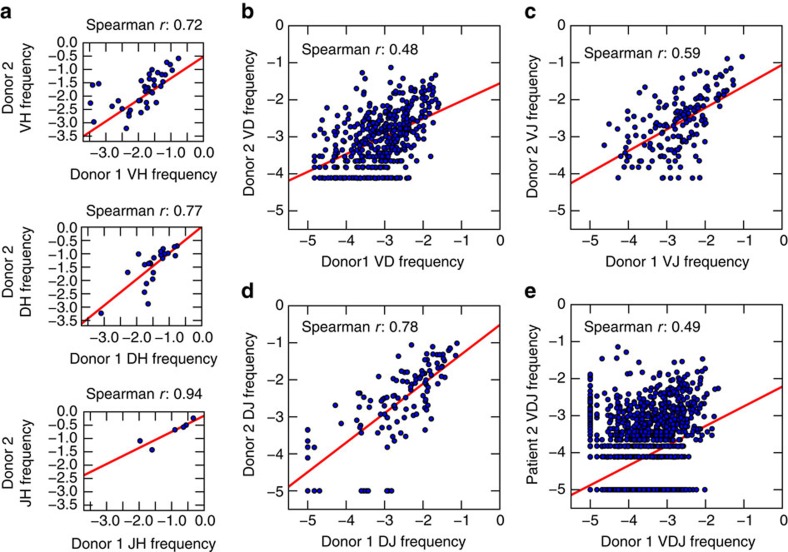
Gene and gene combination use frequencies correlate between Donor 1 and Donor 2. (**a**) Spearman's rank correlation of individual gene frequencies between the two donors: IGHV (top), IGHD (centre) and IGHJ (bottom). (**b**–**e**) Spearman's rank correlation of combination gene frequencies between the two donors: V-D (**b**), V-J (**c**), D-J (**d**) and V-D-J (**e**). (**a**–**e**) Red lines indicate least squares regression.

To verify our longitudinal observations of stability and random gene choices from Donor 1, we analysed a second donor across two years (Fig. 6). We identified 38 IGHV genes, 22 IGHD genes and 6 IGHJ genes (5,016 combinations, 6,763 cells, 93,936 reads) (Supplementary Table 1 and Supplementary Fig. 6). Donors 1 and 2 show highly correlated IGHV gene usage (*r*=0.82). Thus, the trends observed in Donor 1 are also observed in Donor 2. Specifically, individual IGHV, IGHD and IGHJ gene usages are time stable (Supplementary Fig. 6), as are the gene combinations (Supplementary Fig. 7). Consistent with Donor 1, Donor 2 shows no preferential pairing in gene combinations (Supplementary Fig. 8). These results are highly consistent with the trends observed in Donor 1, and together, they indicate that BM plasma cell antibody gene and gene combination usage show surprisingly minimal variation between individuals and across time. Interestingly, minimal variation and a high degree of correlation is maintained when the BM plasma cell repertoires of Donor 1 or Donor 2 are compared with the BM plasma cell repertoire that was obtained from a single donor (age 64) in a separate study^5^ (Supplementary Figs 9 and 10).

Like Donor 1, no persistent CDR-H3 clonotypes are found among 24,287 mBCs sorted from the same biopsies across 2.3 years in Donor 2. In contrast, persistent CDR-H3 clonotypes (165) are readily detected in the BM plasma cell compartment (Supplementary Fig. 12). Importantly, these 165 clonotypes are exclusive to the plasma cell compartment (that is, absent among mBCs). Lastly, as a measure of the quality and integrity of the B-cell sequence data sets derived from the two donors, we observe no inter-donor sequences shared between their mBC compartments, as expected, and only one of the total 188 persistent plasma cell clonotypes is common between the two donors.

## Discussion

Next-generation sequencing has enabled unprecedented ability to explore the details of the human B-cell repertoire^30^,^31^. Whereas previous studies have been able to describe some aspects of the B-cell repertoire at a single point in time, our study harnesses the power of next-generation sequencing and longitudinal biopsies of BM to elucidate the temporal dynamics of BM plasma cells over 6.5 years. Importantly, our data provide molecular resolution of antibody identity in the form of CDR-H3 clonotypes, which is not possible with classic techniques like enzyme-linked immunosorbent assay.

In this study, we show that the human plasma cell compartment is naturally polarized in both IGH gene choice and gene combination and that the polarization is maintained over time. Although our donors were originally diagnosed with and treated for neuroblastoma, they had been asymptomatic and disease-free for several years, and it was during this span when their BM biopsies were acquired; moreover, it is noteworthy that their IGH polarization is statistically similar to a distinct next-generation data set obtained independently by another research group^5^ using a single donor at a single point in time. We also found that the bias is not primarily a result of gene linkage, suggesting there are additional genomic or extrinsic factors that contribute to polarization. Specifically, next-generation sequencing of identical twin pairs^32^,^33^ has revealed clear trends for genetic, or heritable, determinants of IGH gene segment use. Nonetheless, the CDR-H3 region maintains hypervariability and ‘fingerprints' inter-individual variation that distinguishes twin pairs. In addition, the long arms race between the human immune system and the antigens it has confronted throughout evolutionary history may have established a preferential gene choice long ago, and thus there may exist common antibody-mediated solutions to protective immunity. Higher expressing genes are likely broad-spectrum antibodies that have been useful in fighting particular classes of disease and continue to do so today. For example, IGHV1-69 is repeatedly implicated in next-generation studies of anti-viral antibody repertoires (for example, influenza and HIV-1), and the ‘inherently autoreactive' IGHV4-34 element is associated with a range of autoimmune disorders (for example, cold agglutinin disease and systemic lupus erythematosus). Indeed, convergent, or ‘public', responses using these IGHV gene segments coupled within homologous CDR-H3 clonotypes continue to be discovered^34^,^35^,^36^.

Immunological memory is a well-established concept, and mBCs and BM plasma cells are thought to be key contributors, in part, through their putative cellular longevity and hypothesized capacity for self-renewal. How intrinsic longevity might be established and maintained remains an outstanding question. It has been proposed that mBCs generate plasma cells for the lifetime of the human host. It is further hypothesized that mBCs are endowed with a stem cell-like capacity for self-renewal and could be the basis for the continual production of plasma cells^37^. Evidence in support of this hypothesis includes the demonstration that polyclonal activation of mBCs results in their differentiation into plasma cells *in vitro*^14^. Since class-switched mBCs coexist with plasma cells in human BM^38^, we sequenced both compartments to test the hypothesis that BM mBCs may be a renewable source of plasma cells and, indirectly, the source of long-term antibody production in humans. Steady usage of IGH gene and gene combinations observed in both donors throughout our experiment suggests that there are large resident pools of plasma cells of the same identity, from which we can sample continuously with no loss of relative expression levels. Most importantly, we observe years-long temporal persistence of 188 unique, highly diverse CDR-H3 clonotypes exclusively within the plasma cell compartment, whereas CDR-H3 temporal persistence was devoid in the mBC compartment. This provides a crucial point of comparison between these two B-cell subsets pivotal to immunological memory. The data imply that the molecular sequence stability in the plasma cell compartment is due to persistence of the cellular clonotype. It is not simply a reflection of the naïve B-cell repertoire nor mBC repertoire in general (that is, heritable influences of IGH gene use, nor replenishment from the mBC compartment).

Whereas ample data have established the persistence of antigen-specific serum Ig titers, which have half-lives of decades or longer^4^, there is to date no insight as to whether the molecular composition of these antibody titers is a homogenous pool of Ig maintained by a handful of long-lived plasma cell cellular clonotypes or is rather a continual flux and turnover of transitory plasma cell clonotypes. Although our results are unable to verify the lifespan of any one particular plasma cell, we can conclude that clonal members of the CDR-H3 clonotype, which defines identity and binding specificity at the molecular level, does persist for at least 6.5 years. Our results suggest that clonotype persistence contributes to the mechanism underlying long-term immunological memory.

In conclusion, we use high-throughput, next-generation sequencing to definitively identify long-term persistent BM plasma cell clonotypes, which has implications in clinical intervention studies, vaccines and immunotherapy. Future next-generation sequencing studies can provide an even more detailed picture of the B-cell immune repertoire including advances in VH:VL native-pair sequencing (paired BCR-Seq (refs 22, 39)), analysis of correlations between BM plasma cell repertoires and serum Ig species (Ig-Seq (refs 20, 21, 31)), and examination of the connectivity of B cells at various developmental stages (for example, clonal relationships between circulating mBCs and sessile BM plasma cells). Our study provides a foundation upon which these future studies can be built.

## Methods

### Ethical approval

All procedures were performed with informed parental consent at the Memorial Sloan-Kettering Cancer Center under a protocol approved by the MSKCC Institutional Review Board. The protocol is registered at ClinicalTrials.gov (NCT00588068).

### Bone marrow specimens

Serially acquired human BM specimens were collected from two donors by aspiration from the ileac crest, and mononuclear cells were enriched by Ficoll hypaque centrifugation. The two adolescent–teenage donors (10–17 years of age) were originally diagnosed with neuroblastoma but had been asymptomatic and disease-free for many years according to routine BM histology before the first time points in our study. A complete description of the donors' past medical history and ages at the time of the multiple time point collections is included in Supplementary Table 1. Aspirates were withdrawn from four sites and combined (total of 8–10 ml from 4 sites, 2–2.5 ml per site) drawn from the following: anterior right iliac crest, anterior left iliac crest, posterior right iliac crest and posterior left iliac crest. The same attending physicians performed these procedures and usually biopsied through the same surgical site each time. De-identified specimens were shipped overnight on dry ice to the University of Texas at Austin.

### Flow cytometry and isolation of plasma cells

BM samples were quick-thawed in a 37 °C H_2_O bath and slowly diluted into RPMI-1640 complete medium containing DNaseI (Sigma D 4513; 20 U ml^−1^), pelleted, washed and re-suspended in 2 ml FACS buffer (Dulbecco's phosphate buffered saline+0.5% bovine serum albumin Fraction V). Cell viability was determined using Trypan Blue exclusion and on average was approximately 90% per specimen. After a 1-h recovery at room temperature, BM cells were stained for 30 min at room temperature using empirically determined optimal titrations of monoclonal antibodies: CD38-FITC (HIT2), CD138-PE (B-B4), CD27-APC (M-T271) and CD19-v450 (HIB19). CD19^+/–^CD38^++^CD138^+^ cells in human BM were collected as plasma cells. Plasma cells were observed to be heterogeneous for expression of the CD19 B-lineage marker; therefore, CD19-gating was avoided. CD38^++^CD138^+^ plasma cells were additionally gated by forward and side light scatter properties (FSC versus SSC) to exclude debris, apoptotic cells and remnant granulocytes. In a subset of BM specimens, mBCs were also collected as CD19^+^CD27^+^CD38^–^CD138^–^. Donor 1 included mBCs at 0, 0.5, 0.6, 1.5 and 4.0 years; Donor 2 included mBCs at 0 and 2.3 years. All cell sorts were performed on a FACSAria (BD Biosciences). Cells were sorted directly into TRI Reagent for RNA preservation.

### RT-PCR and high-throughput sequencing of IGH genes

Total RNA was isolated using the RNeasy Micro Kit (Qiagen). Approximately 100 ng of total RNA was then used to prepare oligo-dT primed cDNA using the SuperScript III First-Strand Synthesis System (Thermo-Fisher Scientific) according to the manufacturer's protocol. Approximately 25–50% (5–10 μl) of cDNA was then used as template for PCR amplification of variable genes (recombined VHDJH region, which encodes the V region) of IGH isotypes IgM, IgG and IgA. PCR primers have been published^18^. FastStart High Fidelity PCR System (Roche) was used for amplification combining the following thermocycler conditions: 92 °C denaturation for 3 min; 92 °C 1 min, 50 °C 1 min, 72 °C 1 min for 4 cycles; 92 °C 1 min, 55 °C 1 min, 72 °C 1 min for 4 cycles; 92 °C 1 min, 63 °C 1 min, 72 °C 1 min for 20 cycles; and a final extension of 72 °C for 7 min. Samples were then submitted to the University of Texas Genome Sequencing and Analysis Facility for library construction using the NEBNext Quick DNA Library Kit for 454 (New England Biolabs) and next-generation sequencing was accomplished using the Roche 454 GS FLX technology using titanium long-read chemistry. Read counts per sample are listed in Supplementary Table 1.

### Data processing and visualization

IGHV, IGHD, IGHJ and CDR-H3 regions for each read was quality filtered, processed and annotated using the VDJFasta utility^40^. Reference IGHV, IGHD and IGHJ genes from the international ImMunoGeneTics database^41^ were used. Mann-Kendall tests were performed in Matlab, against the null hypothesis of no trend (alpha=0.05). Spearman *r* non-parametric correlation analysis was performed in Python using the scipy library. CDR-H3 sequences were clustered to form antibody clonotypes, as established previously^25^,^42^, using full-length VHDJH gene nucleotide sequences. VHDJH genes were grouped into clonotypes based on single-linkage hierarchical clustering, and cluster membership required ≥85% identity across the CDR-H3 amino sequence (as measured by Levenshtein edit distance).

Circular visualization plots were created with Circos software v0.67-7 (ref. 43) where genes were sorted by expression within each time point and connected to adjacent time points via coloured lines showing their expression levels. All other data visualization was performed using Python and matplotlib.

### Data availability

All sequence data have been deposited to NCBI SRA under BioProject accession code PRJNA310043.

## Additional information

**How to cite this article:** Wu, G. C. *et al*. Temporal stability and molecular persistence of the bone marrow plasma cell antibody repertoire. *Nat. Commun.* **7,** 13838 doi: 10.1038/ncomms13838 (2016).

**Publisher's note:** Springer Nature remains neutral with regard to jurisdictional claims in published maps and institutional affiliations.

## Supplementary Material

Supplementary Information Supplementary Figures and Supplementary Tables.
