# Supplementary material for: Temporal stability and molecular persistence of the bone marrow plasma cell antibody repertoire
Source: Nat Commun. 2016 Dec 21;7:13838. doi: 10.1038/ncomms13838 (PMC5187582; doi:10.1038/ncomms13838)
Supplement: Supplementary Information — Supplementary Figures and Supplementary Tables. [file ncomms13838-s1.pdf]

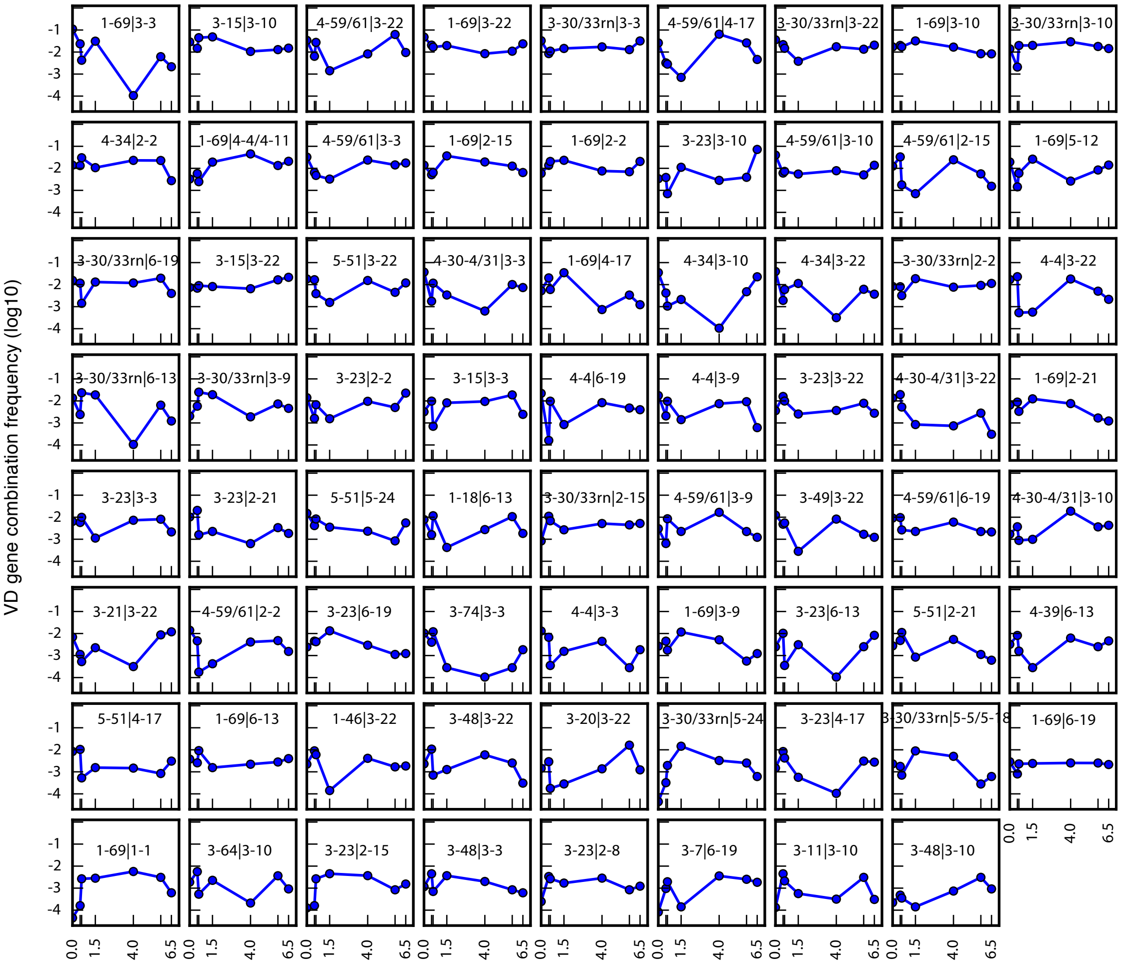

Supplementary Figure 1. IGH V-D combination gene use frequency of plasma cells from Donor 1. Plots are sorted by decreasing mean frequency. Only gene identifications that appear in all timepoints are shown.

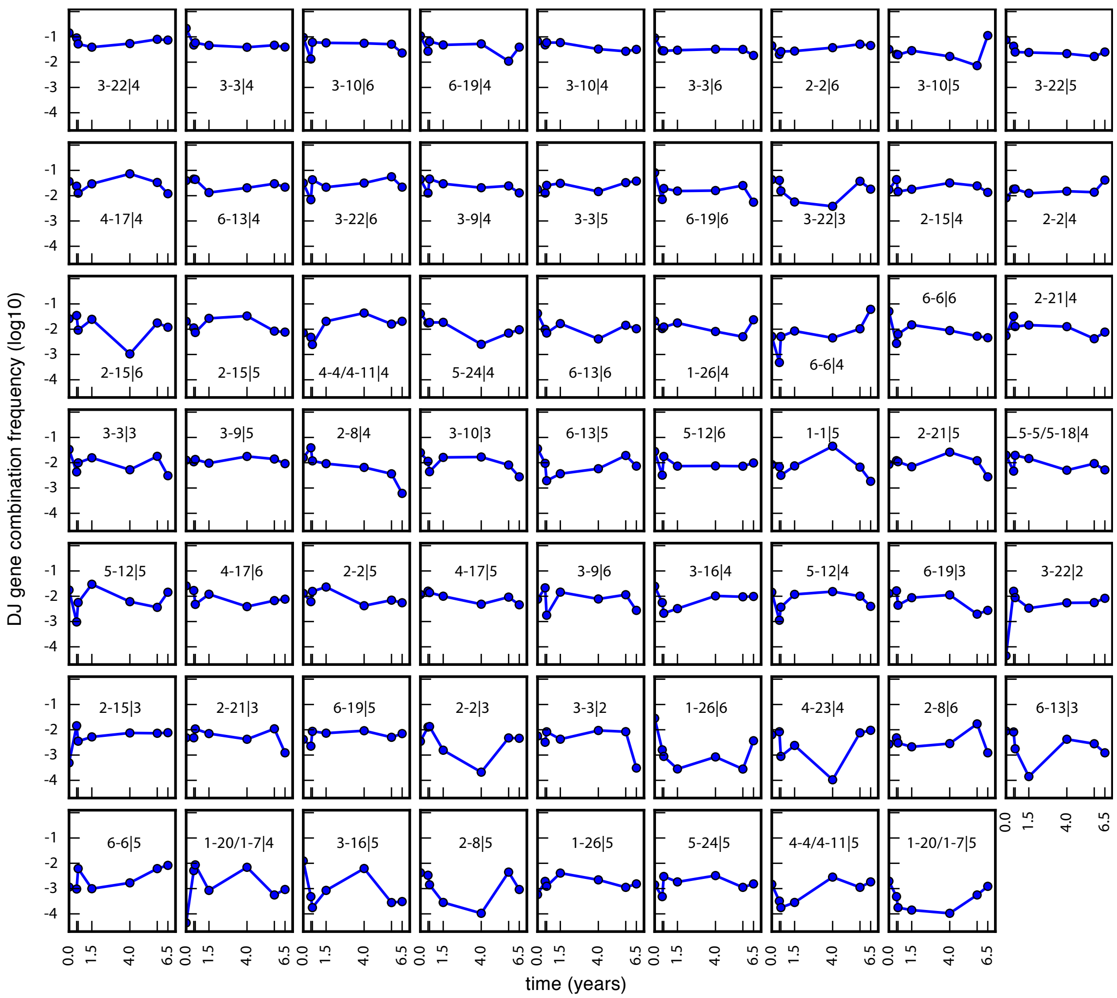

Supplementary Figure 2. IGH D-J usage frequencies for Donor 1 are shown. Plots are sorted by decreasing mean frequency. Only gene identifications that appear in all timepoints are shown.

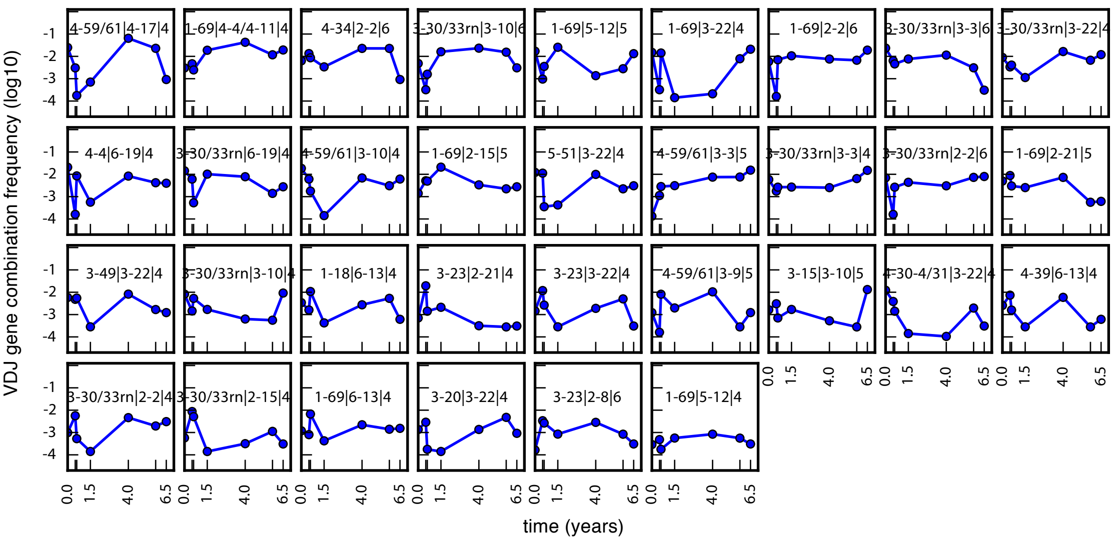

Supplementary Figure 3. IGH V-D-J usage frequencies for Donor 1 are shown. Plots are sorted by decreasing mean frequency. Only gene identifications that appear in all timepoints are shown.

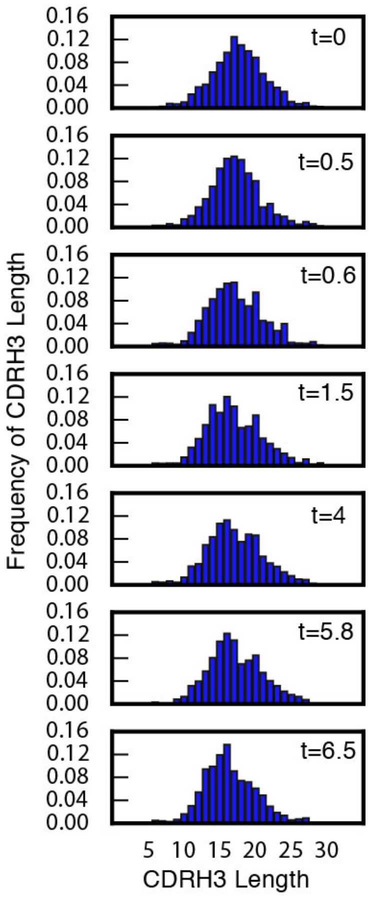

Supplementary Figure 4. CDR-H3 length distribution for each timepoint from Donor 1.

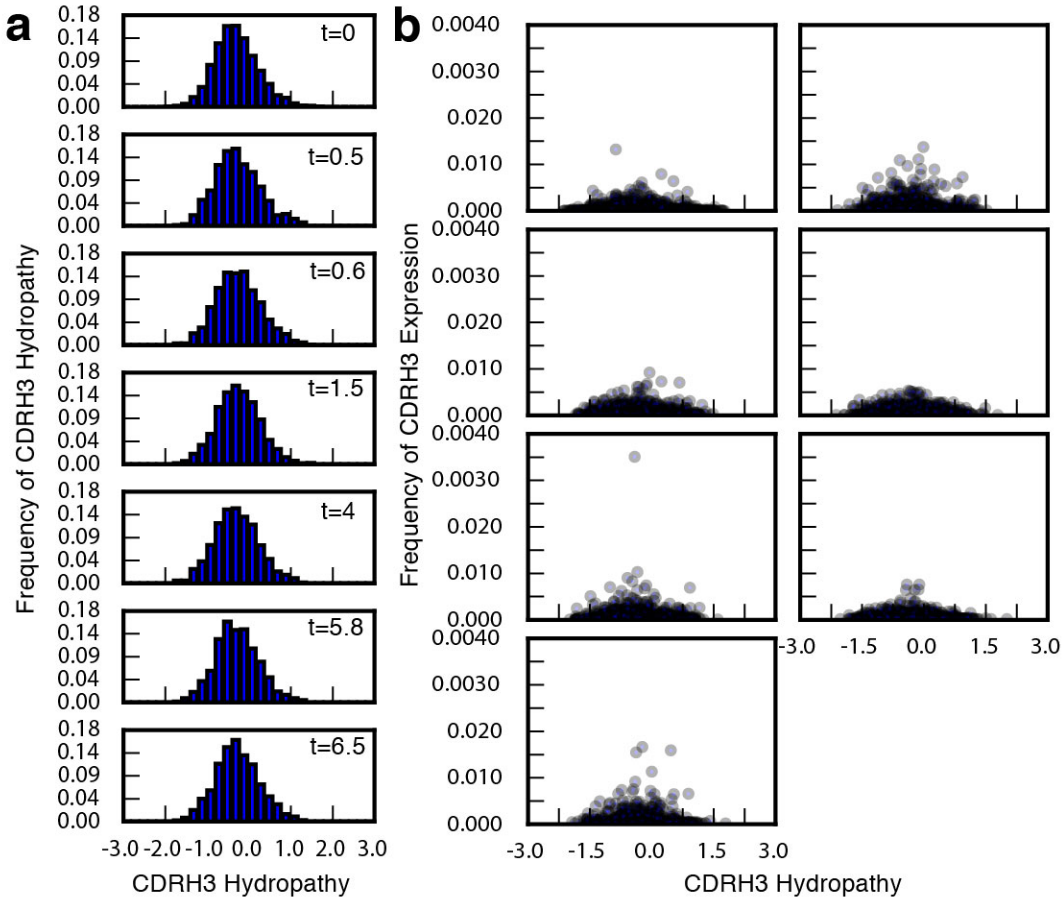

Supplementary Figure 5. For Donor 1: (a) CDR-H3 hydropathy distribution for each timepoint. (b) CDR-H3 frequency versus hydropathy scatter plot.

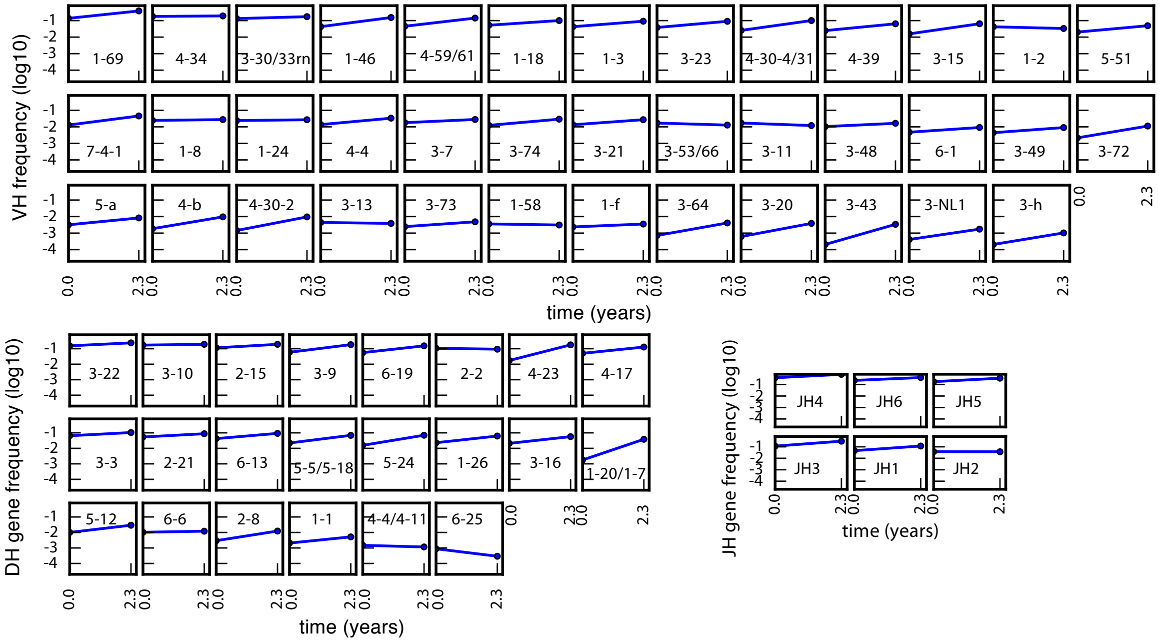

Supplementary Figure 6. For Donor 2: (a-c) IGHV (a), IGHD (b), and IGHJ (c) gene usage frequency over time. Plots are sorted by decreasing mean frequency. Only gene identifications that appear in all timepoints are shown. (d) Mean frequency of IGHV gene use. Error bars are standard deviation.

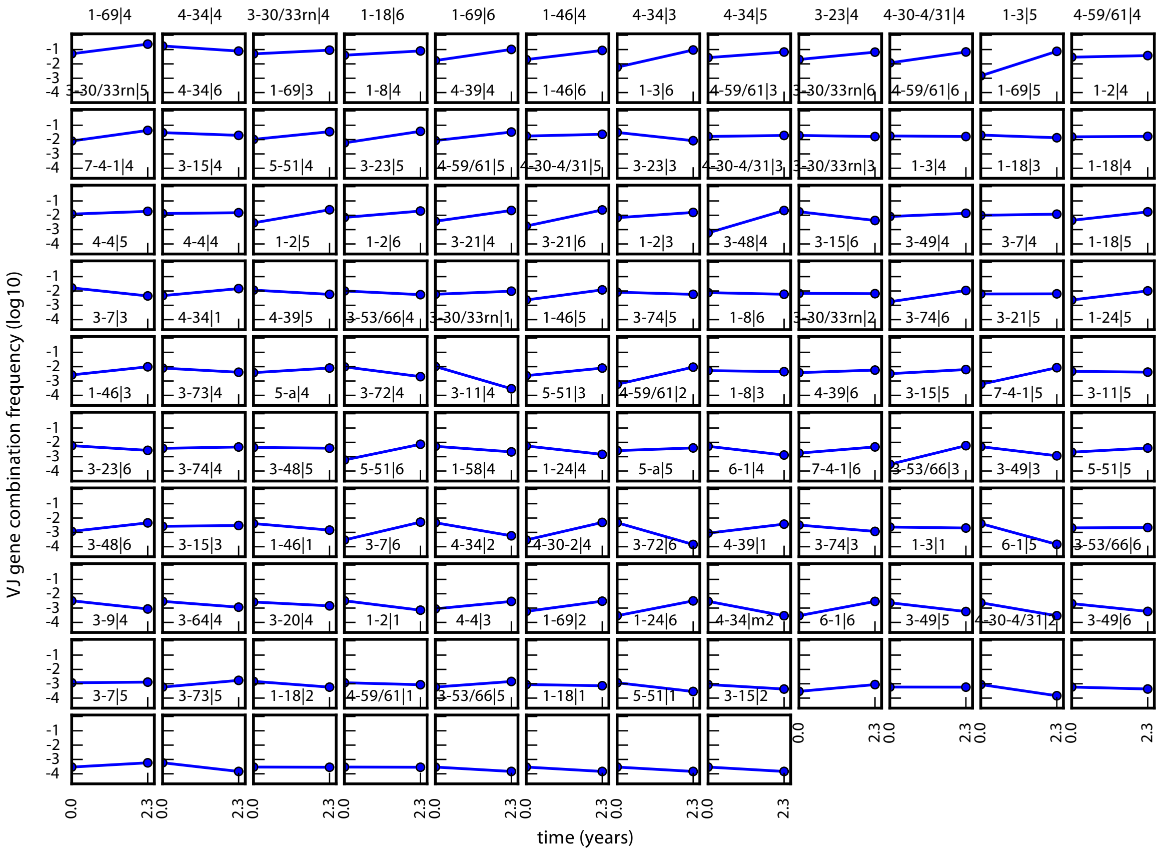

Supplementary Figure 7. IGH V-J usage frequencies for Donor 2 are shown. Plots are sorted by decreasing mean frequency. Only gene identifications that appear in all timepoints are shown.

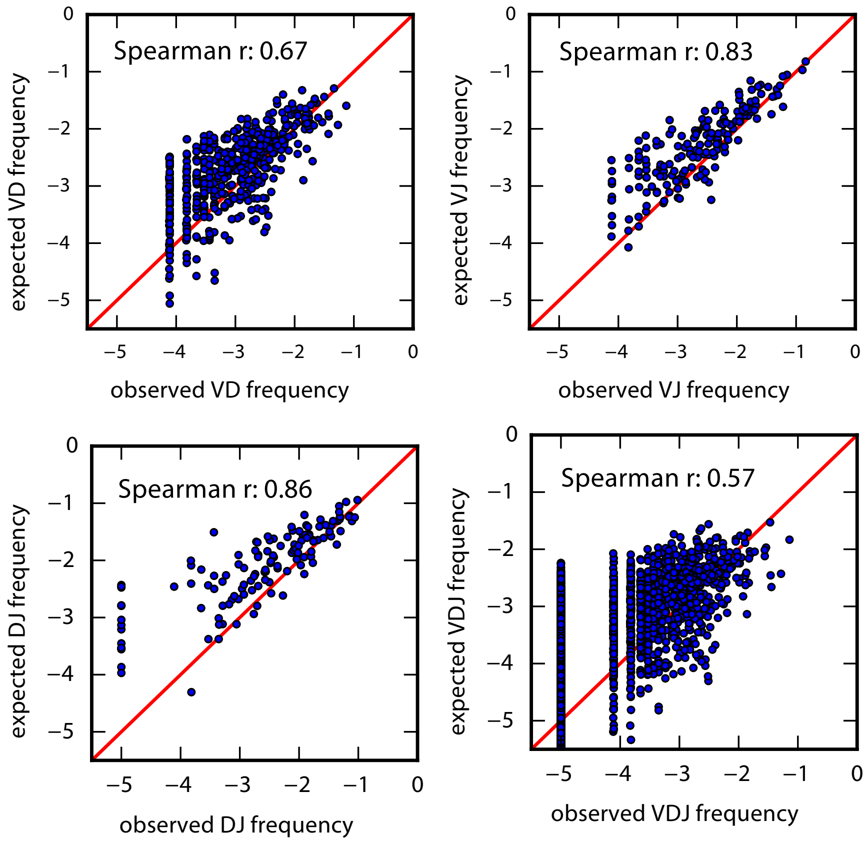

Supplementary Figure 8. Gene combinations among BM plasma cells are randomly assorted in Donor 2. (a-d) Spearman's rank correlation of expected versus observed IGH V-D (a), V-J (b), D-J (c), and V-D-J (d) gene combination frequencies. Expected (by random association) frequencies are calculated as products of the frequencies of the individual component genes. Diagonal lines in red indicate no difference between the expected and observed frequencies.

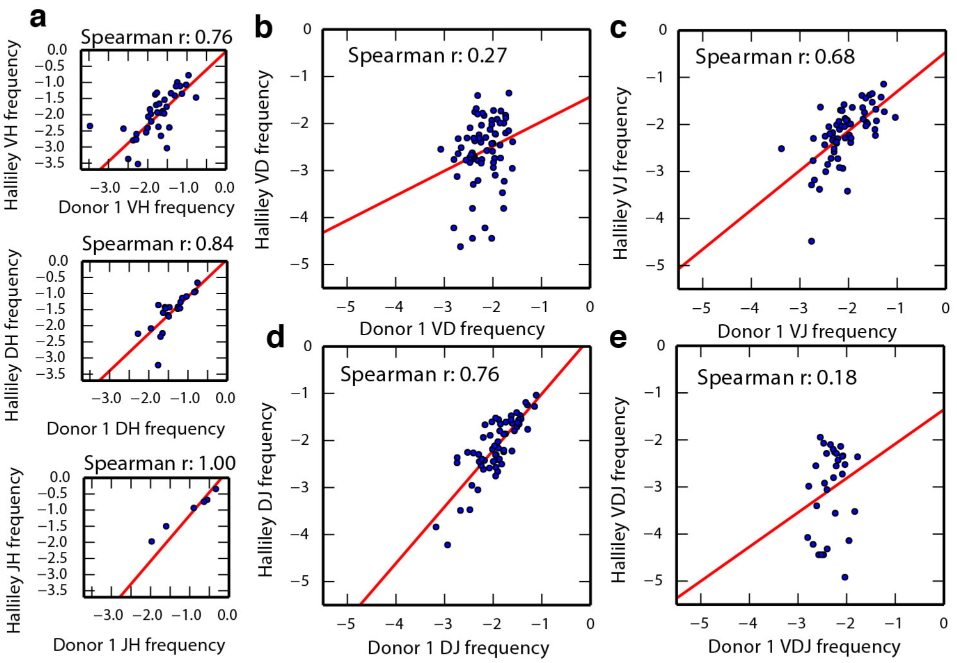

Supplementary Figure 9: Gene and gene combination use frequencies correlate between Donor 1 and donor from Halliley, 2015. (a) Spearman's rank correlation of individual gene frequencies between the two donors: IGHV (top), IGHD (center), and IGHJ (bottom). (b-e) Spearman's rank correlation of combination gene frequencies between the two donors: V-D (b), V-J (c), D-J (d), and V-D-J (e). (a-e) Red lines indicate least squares regression.

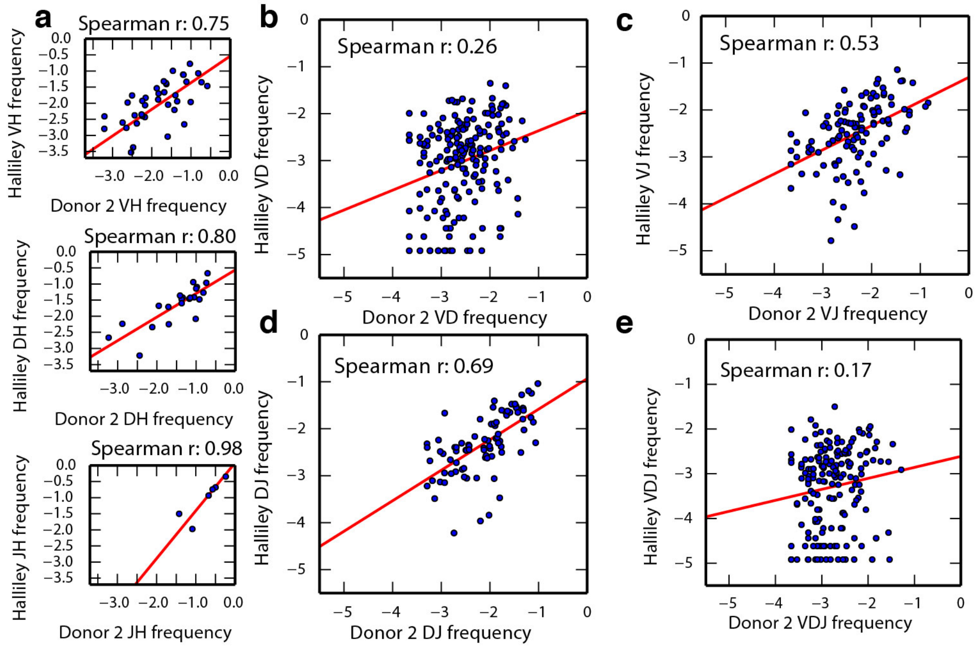

Supplementary Figure 10. Gene and gene combination use frequencies correlate between Donor 2 and donor from Halliley, 2015. (a) Spearman's rank correlation of individual gene frequencies between the two donors: IGHV (top), IGHD (center), and IGHJ (bottom). (b-e) Spearman's rank correlation of combination gene frequencies between the two donors: V-D (b), V-J (c), D-J (d), and V-D-J (e). (a-e) Red lines indicate least squares regression.

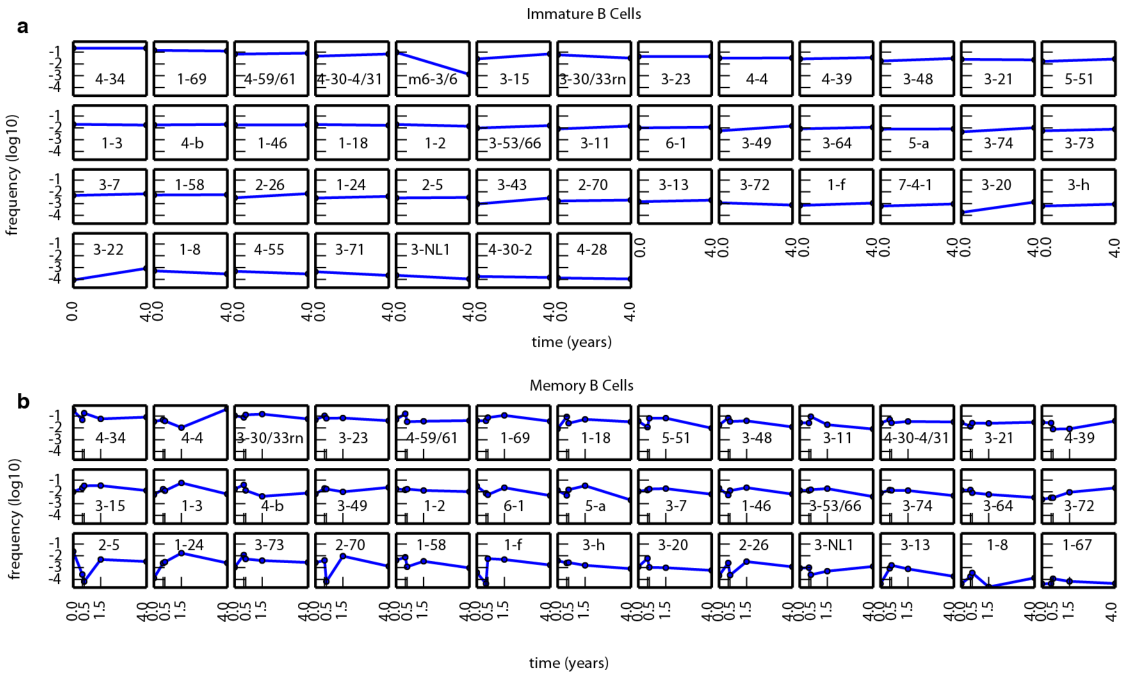

Supplementary Figure 11. IGHV frequencies across four years in Donor 1 in immature B and memory B cell subsets isolated from bone marrow. Plots are sorted by decreasing mean frequency.

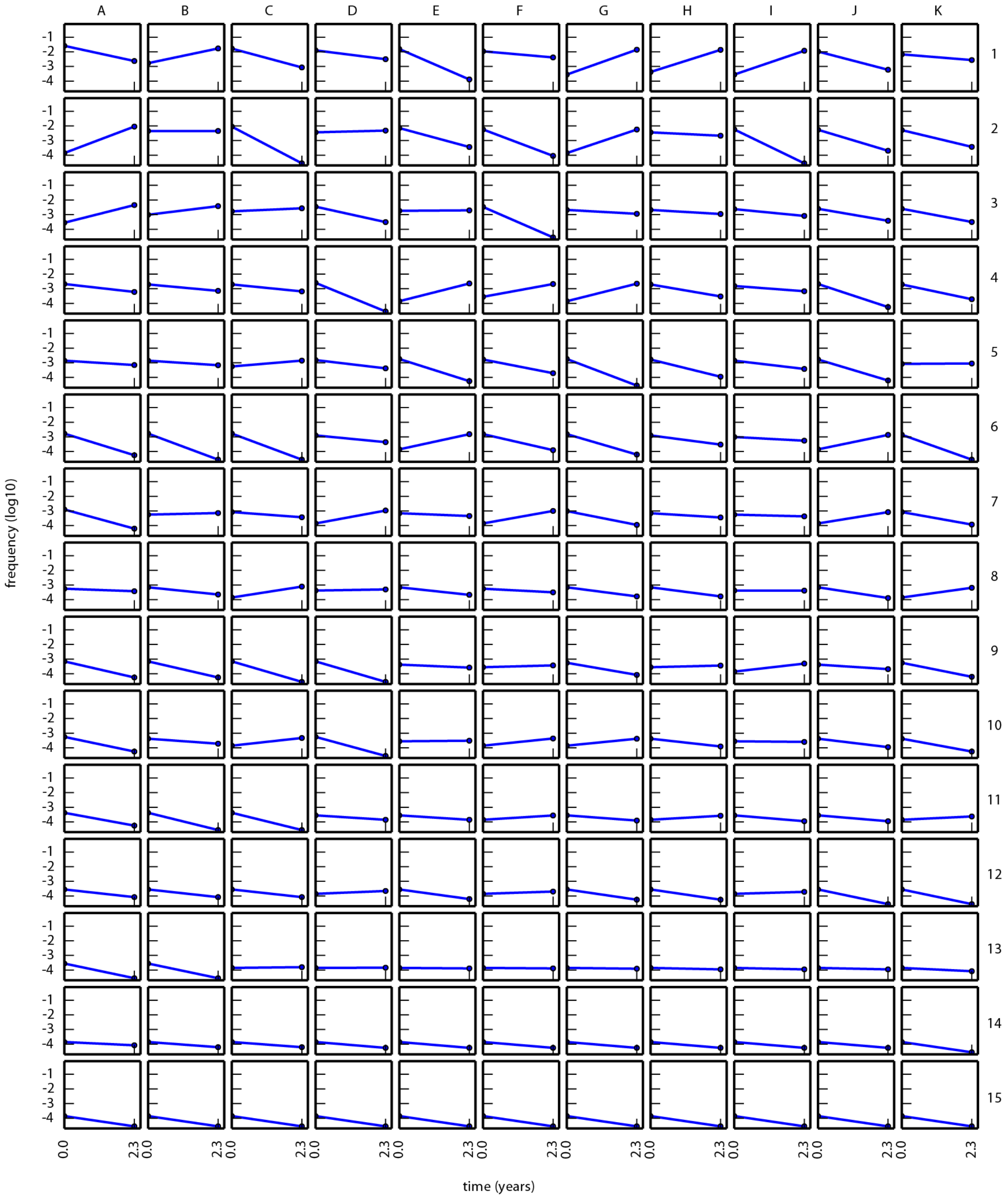

Supplementary Figure 12. Gene usage frequency over time of the 165 persistent clonotypes found in both timepoints in Donor 2. Plots are sorted by decreasing mean frequency. The gene names (for IGHV and IGHJ), representative amino acid sequences, and isotype information can be found in Supplementary Table 2.

Sample ID	Donor	Age (years)	Time (years)	Cells counted	Read counts	Unique CDRH3s
d1t00a	1	10.9	0	6,674	49,742	4,290
d1t00b	1	10.9	0	1,298	110,619	4,338
d1t05	1	11.2	0.5	2,877	50,468	2,773
d1t06	1	11.5	0.6	5,047	39,683	3,265
d1t15	1	12.4	1.5	5,629	45,949	3,691
d1t40	1	14.9	4	1,870	70,692	3,709
d1t58	1	16.5	5.8	14,307	20,330	3,276
d1t65	1	17.3	6.5	6,735	21,996	2,843
d2t00	2	13.5	0	3,642	17,726	2,120
d2t23a	2	15.78	2.28	2,021	39,096	2,855
d2t23b	2	15.78	2.28	1,100	37,114	4,999
Total				51,200	503,415	38,159

Supplementary Table 1. Donor history and sequencing Information. Bone marrow (BM) plasma cells were isolated from each sample by flow cytometry. BM plasma cells are defined as CD138+ CD38++ cells from bone marrow mononuclear cells. See **Fig. 1** and Methods. Donor 1 was diagnosed at the age of 9 years with adrenal neuroblastoma metastatic to the bone marrow. Patient underwent multiagent chemotherapy consisting of high dose alkylators, then consolidated with myeloablative therapy followed by hematopoietic stem cell transplant. Because of progressive disease in bone marrow and bones at age 10, local radiation and systemic 131 I-MIBG was given followed by anti-GD2 antibody immunotherapy, 3F8+ GM-CSF+ beta-glucan+ 13-cis- retinoic acid till age 14. Patient continued in remission through age 17 years. Because of cancer therapy, patient had to be re-immunized with tetanus, *Hemophilus influenza b* (Hib), Hepatitis B, and Polio at age 12 (before sample d1t15) and boosted again with Hib, Hepatitis B and Polio at age 13 (between sample d1t15 and d1t40). MMR (mumps measles rubella) vaccine was then given at age 14 (before sample d1t40 and d1t58 and d1t65). Donor 2 was diagnosed at the age of 4 with mediastinal neuroblastoma metastatic to bone and bone marrow and received high dose multiagent chemotherapy. Tumor recurred as epidural mass in the lumbar at the age of 12 and was retreated with high dose multiagent chemotherapy followed by myeloablative therapy plus autologous hematopoietic stem cell rescue and focal radiation to the spine. Patient was treated with anti-GD2 3F8 immunotherapy plus oral etoposide till age 14, and remained in remission through age 20 years.

Position	VH Gene	JH Gene	Isotype	Representative CDRH3
A1	IGHV1-69	IGHJ1	IGHG	CARHPSNSWFRIHFQHW
B1	IGHV1-69	IGHJ1	IGHA	CARGGEQGNYYRTWEYYPYW
C1	IGHV4-34	IGHJ4	IGHM	CARWIRYCSGGDCYPSMYYFDYW
D1	IGHV1-18	IGHJ6	IGHG	CARDRCSGGSCYPGRPQYFYGMDVW
E1	IGHV1-24	IGHJ3	IGHG	CATVAITVDYDSTAYDGLDVW
F1	IGHV1-69	IGHJ4	IGHG	CAKASQNYDSSGYFDCW
G1	IGHV1-3	IGHJ6	IGHA	CARVTATSILGDSGRHHYYAMDVW
H1	IGHV1-46	IGHJ3	IGHA	CARGLRGNLRVLAILPAGAFDMW
I1	IGHV1-46	IGHJ6	IGHA	CARPLSQRGHFYYGMDVW
J1	IGHV4-34	IGHJ4	IGHG	CARGRIVVAPAAMFRRRGSDYFDYW
K1	IGHV1-69	IGHJ6	IGHG	CASDNKIYDYGDGDFQYHNLAVW
A2	IGHV3-15	IGHJ5	IGHG	CVTQATAATAGLAAIITNFDLW
B2	IGHV1-46	IGHJ3	IGHA	CARVIKPGKNDVFEIW
C2	IGHV1-3	IGHJ5	IGHG	CARVVDTPFCRSSNCHNWLDPW
D2	IGHV1-69	IGHJ5	IGHA	CATWGGHCTWYNWCSRVTAFSLDIW
E2	IGHV3-23	IGHJ4	IGHA	CAKAPLDVVTELDYW
F2	IGHV4-34	IGHJ4	IGHG	CARVVNGVAPAAIFHRRGLDYFDYW
G2	IGHV1-69	IGHJ3	IGHA	CARDLRDMSASGGVTFDAFNIW
H2	IGHV1-69	IGHJ4	IGHA	CARWDGHCSFFNWCSGRTVFPLDFW
I2	IGHV1-8	IGHJ4	IGHG	CARGGGSNWRRIHPVDYW
J2	IGHV1-69	IGHJ5	IGHG	CARDMNDYYDPSGYSGALDHW
K2	IGHV4-34	IGHJ4	IGHG	CARARVRNPTGLFRRGYPVFDSW
A3	IGHV4-30-4/31	IGHJ4	IGHA	CAVMYNWNYGFDYW
B3	mIGHV6-3/6	IGHJ4	IGHG	CARYVWYSSYPHSYSGLDYW
C3	IGHV4-30-4/31	IGHJ3	IGHG	CARVGYDGRDYVGKYGFDIW
D3	IGHV4-34	IGHJ4	IGHG	CAGKRRLYSYGLGSYYYFESW
E3	IGHV3-7	IGHJ5	IGHM	CARRGPTFWSGYYESYYDAW
F3	IGHV1-3	IGHJ6	IGHG	CATTNRQIRAARDFYGMDVW
G3	IGHV3-53/66	IGHJ4	IGHG	CARTGQDWYDIHLEHW
H3	IGHV3-30/33rn	IGHJ4	IGHG	CARELYAGSSGYVGYFDSW
I3	IGHV1-69	IGHJ4	IGHA	CATWGGQCAWYNWCNRNTAFSLDFW
J3	IGHV1-69	IGHJ5	IGHG	CALGVKGFMVHGGAKNWFESW
K3	IGHV1-18	IGHJ3	IGHG	CARGTDYGDYIGAFDFW

Supplementary Table 2. The gene names (for IGHV and IGHJ), representative amino acid sequences, and isotype information for Supplementary Fig. 12 positions A1 to K3.

Position	VH Gene	JH Gene	Isotype	Representative CDRH3
A4	IGHV1-3	IGHJ6	IGHG	CARVTATSELRDTGRHHYYIMDVW
B4	IGHV3-15	IGHJ6	IGHG	CATGSHPGRKFYYGSVFW
C4	IGHV3-30/33rn	IGHJ6	IGHG	CARDSVHMINSYDYYFGMDVW
D4	IGHV3-30/33rn	IGHJ4	IGHG	CARDCSGYFCFDHW
E4	IGHV4-34	IGHJ3	IGHG	CAACGGSSSCGRAFDIW
F4	IGHV5-51	IGHJ4	IGHG	CARHRGDPFYHGLESRMRFFDYW
G4	IGHV4-34	IGHJ6	IGHG	CARGHDFLSPPGYYYGLDVW
H4	IGHV1-69	IGHJ3	IGHG	CARTRALADGGAFEIW
I4	IGHV3-30/33rn	IGHJ6	IGHG	CAKEESNHVNYYYYYAMDVW
J4	IGHV3-30/33rn	IGHJ5	IGHG	CARYYYDTSGPVLDLW
K4	IGHV4-34	IGHJ4	IGHG	CARLVSVVVPSALFHRRGLEYFDSW
A15	IGHV4-34	IGHJ3	IGHG	CARRVATIARGAFDIW
B5	IGHV3-30/33rn	IGHJ4	IGHG	CARIHISAPGNNFDYW
C5	IGHV1-24	IGHJ6	IGHA	CATGEGDAYNYGLDVW
D5	IGHV3-30/33rn	IGHJ1	IGHG	CARIHIAAHGNNFESW
E5	IGHV4-34	IGHJ4	IGHG	CASFAGFRDKWSHLAYW
F5	IGHV1-18	IGHJ4	IGHG	CARDLKGVSVSATFWGLSDDW
G5	IGHV3-11	IGHJ4	IGHG	CARVHSYGDRGPFDYW
H5	IGHV4-34	IGHJ6	IGHG	CVRGHPYKGLGKLYYHYYYGMDVW
I5	IGHV1-69	IGHJ5	IGHA	CATWGGHCTWYNWCSRVTAFSLDIW
J5	IGHV1-46	IGHJ6	IGHG	CARGDTMVGGIDCMDVW
K5	IGHV1-69	IGHJ6	IGHG	CSRSLRGRWLQSDRDYYYAMDVW
A6	IGHV4-30-4/31	IGHJ4	IGHG	CARVVETATDYW
B6	IGHV3-30/33rn	IGHJ4	IGHA	CARVFESYNLDHW
C6	IGHV4-59/61	IGHJ2	IGHG	CARGRSGDYILYWYLDLW
D6	IGHV1-24	IGHJ5	IGHG	CASIMGHDYGDYVETPNWFDPW
E6	IGHV1-46	IGHJ6	IGHA	CARDPVGATRGGGGMDVW
F6	IGHV1-2	IGHJ3	IGHG	CARGSDRGYAVLGELSAGGAFDIW
G6	IGHV1-2	IGHJ5	IGHM	RATTYCNGVCPDDNWFDPW
H6	IGHV1-2	IGHJ5	IGHG	CARDGRPLQFLKNWFDPW
I6	IGHV4-34	IGHJ6	IGHG	CARMVVKQQLLPRFQVGYYGMDVW
J6	IGHV1-18	IGHJ1	IGHA	CTRDNSNYPEYFQHW
K6	IGHV1-8	IGHJ3	IGHM	CARGSYYDSSGHYHRIAFDIW

Supplementary Table 2 (cont.) The gene names (for IGHV and IGHJ), representative amino acid sequences, and isotype information for Supplementary Fig. 12 positions A4 to K6.

Position	VH Gene	JH Gene	Isotype	Representative CDRH3
A7	IGHV3-30/33rn	IGHJ6	IGHG	CARWAYEGTDVYYYYGMDVW
B7	IGHV1-18	IGHJ5	IGHA	CAKDLWTVTPSFNWFDSW
C7	IGHV1-46	IGHJ4	IGHA	CAREFLGPDYYGSGTKYEYW
D7	IGHV1-69	IGHJ6	IGHA	CARVPYFGSGSYYENYYDMDVW
E7	IGHV1-69	IGHJ6	IGHA	CARLPFFGSGSYYENYYDMDVW
F7	IGHV1-69	IGHJ6	IGHG	CAREGGYCTSPRCYVLEWPRNAGPDYYYNYHMNVW
G7	IGHV1-3	IGHJ5	IGHG	CARSDQWLVGLDPW
H7	IGHV4-34	IGHJ6	IGHG	CARGRFKVVVFGVALEYGLDVW
I7	IGHV1-69	IGHJ4	IGHA	CATTEDGRVPGYFDYW
J7	IGHV3-30/33rn	IGHJ6	IGHG	CAKDEQMTATYYYYFYGMDVW
K7	IGHV1-69	IGHJ4	IGHA	CVRESRKDGYGRDW
A8	IGHV4-34	IGHJ6	IGHG	CARRYDASGSHYYFYYYHMDVW
B8	IGHV3-30/33rn	IGHJ4	IGHG	CAKDGGIGFTDFDSW
C8	IGHV1-46	IGHJ4	IGHA	CAREGTSRFFQYW
D8	IGHV3-30/33rn	IGHJ1	IGHG	CARIHIRAGGNNFDSW
E8	IGHV4-30-4/31	IGHJ4	IGHG	CARVGPFDTTGYYFDYW
F8	IGHV1-2	IGHJ4	IGHG	CAREAPNLRYYFDFW
G8	IGHV3-30/33rn	IGHJ2	IGHA	CAKDRGISGSYLDWYFDLW
H8	IGHV4-34	IGHJ4	IGHG	CARGVYSGSGSYDYW
I8	IGHV1-69	IGHJ6	IGHA	CAREETEYTTSSLRTTTPYNYGLDIW
J8	IGHV1-46	IGHJ3	IGHA	CARVTKPGKNDVFEIW
K8	IGHV1-18	IGHJ5	IGHA	CARGHIWKELDSW
A9	IGHV1-3	IGHJ6	IGHG	CARDGRGSYGSDFYHSMDAW
B9	IGHV1-69	IGHJ4	IGHA	CARVPTTNILDSGYDYYFDYW
C9	IGHV1-46	IGHJ4	IGHG	CARDISSWHEPRYYFDDW
D9	IGHV1-8	IGHJ5	IGHG	CARVYGVWGVERGLQNQHFDQW
E9	IGHV1-18	IGHJ5	IGHG	CARDTPNYQLLEDFW
F9	IGHV1-18	IGHJ4	IGHA	CTRDTPNYQLLEDYW
G9	IGHV4-39	IGHJ4	IGHG	CTRDSGFYLRMGYW
H9	IGHV3-21	IGHJ4	IGHA	CARGAGGNPVGPTKEPKGGFDYW
I9	IGHV3-30/33rn	IGHJ4	IGHG	CARIHIRAAGNNFDNW
J9	IGHV1-3	IGHJ4	IGHA	CAREGVDMPTVWPIRPSRNYFDSW
K9	IGHV1-69	IGHJ4	IGHA	CARWNGHCSFFNWCSGRTVFPLDFW

Supplementary Table 2 (cont.) The gene names (for IGHV and IGHJ), representative amino acid sequences, and isotype information for Supplementary Fig. 12 positions A7 to K9.

Position	VH Gene	JH Gene	Isotype	Representative CDRH3
A10	IGHV4-34	IGHJ5	IGHG	CARLGVVLPAAMFSRKGGNQFDPW
B10	IGHV4-b	IGHJ4	IGHA	CARGPRTMYNSNYYDYFFDYW
C10	IGHV1-3	IGHJ6	IGHA	CARVTATSIVTDAGRLWYYAMDVW
D10	IGHV1-8	IGHJ4	-	CARGRGAAVVRPETYW
E10	IGHV1-2	IGHJ4	IGHA	CARAWNDVPGGYW
F10	IGHV4-59/61	IGHJ5	IGHG	CARSTLSYCGDSCYPLDSW
G10	IGHV1-18	IGHJ6	IGHG	CVRDIFSTEWTLGYHGMDVW
H10	IGHV4-34	IGHJ5	IGHG	CARLTSVVPAAMFSRMGGDHFDPW
I10	IGHV3-30/33rn	IGHJ3	IGHG	CAREGSGWLAAFDIW
J10	IGHV3-23	IGHJ4	IGHG	CAKKRLVGFLHHFFDSW
K10	IGHV1-69	IGHJ4	IGHM	CARVMEYCSGGSCYEDFDYW
A11	IGHV1-46	IGHJ3	IGHG	CARGVTLYYGESDAGDAFDIW
B11	IGHV1-18	IGHJ5	IGHA	CARDRCITTSCYPWFDPW
C11	IGHV3-53/66	IGHJ6	IGHA	CARAPGLQGGYYYYYGMEVW
D11	IGHV1-18	IGHJ5	IGHA	CARVDFYDLLPGYCKYW
E11	IGHV3-74	IGHJ4	IGHA	CVRSHTGRYDNW
F11	IGHV1-18	IGHJ5	IGHA	CARDLWTVTPSFNWFESW
G11	IGHV1-69	IGHJ5	IGHG	CATWGGHCTWYSWCSRVTAFSLDIW
H11	IGHV4-34	IGHJ6	IGHG	CVRGPREEPAGPSHPRYYFYYSAIDVW
I11	IGHV1-2	IGHJ4	IGHA	CATSLELRVPDDSW
J11	IGHV4-39	IGHJ3	IGHA	CAREDSYKTRNTFDIW
K11	IGHV1-2	IGHJ4	IGHG	CARTLEDYEDYW
A12	IGHV1-69	IGHJ5	IGHG	CARGRDDYKGEVFDHW
B12	IGHV4-34	IGHJ6	IGHG	CARMVIKQQPLPRFQVAYYGMDVW
C12	IGHV4-34	IGHJ4	IGHA	CARGPPGYALDYW
D12	IGHV1-46	IGHJ6	IGHA	CARDFRAILLVRGVLRDYALDVW
E12	IGHV3-23	IGHJ4	IGHG	CAKEDCSSANCYRLDYW
F12	IGHV4-59/61	IGHJ6	IGHA	CARVVTLRVAGSSQYYMDTW
G12	IGHV1-3	IGHJ6	IGHG	CARVTATSRVTDAGRLWFYAMDVW
H12	IGHV4-59/61	IGHJ4	-	CAVNYDSSGYTRGFDSW
I12	IGHV1-69	IGHJ3	IGHG	CARDGGYCSGRACHAYAFDMW
J12	IGHV1-69	IGHJ6	IGHG	CARDIAVSETDYYYFALDVW
K12	IGHV3-30/33rn	IGHJ4	IGHA	CASELTRVAAAGKGNDYW

Supplementary Table 2 (cont.) The gene names (for IGHV and IGHJ), representative amino acid sequences, and isotype information for Supplementary Fig. 12 positions A10 to K12.

Position	VH Gene	JH Gene	Isotype	Representative CDRH3
A13	IGHV4-59/61	IGHJ3	IGHG	CARPIWEPRDAFDIW
B13	IGHV1-3	IGHJ1	IGHA	CARRPYCSGGSCYTGEYFQHW
C13	IGHV1-8	IGHJ5	IGHA	CARGNKPDHTASSLSKNWFDPW
D13	IGHV1-18	IGHJ6	IGHA	CARDDRYSSAWYLGSYYGMDVW
E13	IGHV4-39	IGHJ5	IGHG	CARHYDFVWGTYRDQARNWFDPW
F13	IGHV5-51	IGHJ3	IGHA	CARPEAISGFYAFDVW
G13	IGHV1-69	IGHJ5	IGHA	CARWDGHCSFFNWCSGRTVFPLDFW
H13	IGHV1-69	IGHJ4	IGHA	CASAGGDDIFAVVTYYW
I13	IGHV3-11	IGHJ4	IGHM	CARGLRGYSYGLSDYW
J13	IGHV4-4	IGHJ4	IGHM	RASRRVGATFYW
K13	IGHV1-18	IGHJ4	IGHA	CARVQSNSIFGVFIPYHLDSW
A14	IGHV4-34	IGHJ5	IGHA	CARWIRYCSGGDCYPSMYYFDSW
B14	IGHV1-2	IGHJ6	IGHA	CFRETQRGYGMDVW
C14	IGHV3-15	IGHJ6	-	CATGSHPGRKVLHGSVVW
D14	IGHV1-69	IGHJ4	IGHA	CARESGDGYNPKRAHVFDYW
E14	IGHV1-69	IGHJ3	IGHA	CASHQPKNYYDSSSYRAFDIW
F14	IGHV1-3	IGHJ5	IGHG	CAREPVPHQLLYWFDPW
G14	IGHV4-34	IGHJ4	IGHG	CARGRIVVASAALFRRRGSDYFDYW
H14	IGHV4-34	IGHJ4	IGHA	CARLVSVVQPAALFHRRGLDYIDFW
I14	IGHV1-18	IGHJ1	IGHG	CARGHIWKELDSW
J14	IGHV3-48	IGHJ5	IGHM	CALSRDGYSHKW
K14	IGHV4-59/61	IGHJ6	IGHM	CARRSGGSHYYMDVW
A15	IGHV4-34	IGHJ6	IGHA	CVRGHPYKGFGEKYYLYYYGMDVW
B15	IGHV4-34	IGHJ4	IGHG	CARGQTALKPVVFGVVITRPTNNYFDYW
C15	IGHV3-21	IGHJ4	IGHA	CARDDGDSVAEEYW
D15	IGHV4-34	IGHJ5	IGHG	CARLGVVVPVAMFSRKEGNHFDPW
E15	IGHV1-2	IGHJ6	IGHA	CARDFLPPGQVATIPLWHGMDVW
F15	IGHV4-34	IGHJ6	IGHM	CARGHEDYSNYYYYGMDVW
G15	IGHV1-8	IGHJ6	-	CARVGGPYSIHYMDVW
H15	IGHV1-69	IGHJ6	IGHG	CARDGRGQRPTRHIIIINTDWYLW
I15	IGHV1-69	IGHJ4	IGHG	CARSPVAGAYFFDYW
J15	IGHV1-69	IGHJ1	IGHG	CARGGNRGVIIGPGNTYPYW
K15	IGHV4-30-4/31	IGHJ4	IGHG	CARGAYFYGSGLDYW

Supplementary Table 2 (cont.) The gene names (for IGHV and IGHJ), representative amino acid sequences, and isotype information for Supplementary Fig. 12 positions A13 to K15.
